# Application of neurally adjusted ventilatory assist in ventilator weaning of infants ventilator weaning

**DOI:** 10.1002/brb3.2350

**Published:** 2021-09-14

**Authors:** Shuna Xiao, Chengjiao Huang, Ying Cheng, Zhi Xia, Yong Li, Wen Tang, Buyun Shi, Lijun Wang, Xiaolan Shu, Ying Jiang, Chenguang Qin, Hui Xu

**Affiliations:** ^1^ Department of Pediatric Critical Medicine Maternal and Child Health Care Hospital of Hubei Province Wuhan China; ^2^ Department of Cardiac Surgery The Second Affiliated Hospital of Nanchang University Nanchang China

**Keywords:** continuous positive airway pressure, infants, neurally adjusted ventilatory assist, value and application, ventilator weaning

## Abstract

**Background:**

To analyze the application of neurally adjusted ventilatory assist in ventilator weaning of infants.

**Methods:**

A total of 25 infants (15 boys and 10 girls) who were mechanically ventilated by PICU in Hubei Maternal and Child Health Hospital were selected as the study subjects. After the improvement of the basic disease, regular spontaneous breathing, and the withdrawal of the ventilator, all the children obtained the electrical activity of the diaphragm (EAdi) signal. Then, each child was given CPAP and NAVA mode mechanical ventilation 1 h before the withdrawal of the ventilator. Each detection index was recorded 30 min after each mode of ventilation.

**Results:**

Two of the 25 children were tracheotomized because of respiratory muscle weakness and could not be converted to NAVA mode without the EAdi signal. Hemodynamic indexes were not statistically different between the two groups of CPAP and NAVA. PaCO_2_ is not significantly different in the two modes, and both were at normal levels. The PIP in NAVA mode is lower than that in CPAP mode (*p* < .05), and its EAdi signal was correspondingly low. There were significant differences in the peak pressure (Ppeak), mean pressure (Pmean), and compliance and mean arterial pressure (*p* < .01) between the CPAP and NAVA model ventilation in 23 patients.

**Conclusion:**

NAVA can significantly improve the coordination of patients. The therapeutic effect of NAVA was better, which was beneficial to the prognosis of patients and had positive application value in the withdrawal of ventilators in patients.

## Abstract 2

NAVA ventilation, a new ventilation mode, can maximize human–machine coordination while ensuring that the ventilator supports the appropriate ventilation level for patients, reducing complications related to mechanical ventilation, protecting organ functions, improving respiratory mechanics and hemodynamics.

## 1 BACKGROUND

Mechanical ventilation can improve patient oxygenation, correct carbon dioxide storage, and provide time guarantee for the treatment of primary disease. However, when the underlying disease is controlled, how to assess whether patients have ventilator weaning conditions has become a difficult problem for clinicians. Available data indicate that about 60%–65% of patients admitted to intensive care unit (ICU) for mechanical ventilation survive, about 85% of mechanically ventilated patients are successfully extubated. In comparison 15% or more patients require re‐intubation within approximately 48 h (MacIntyr, 2012). Due to the prolonged duration of mechanical ventilation, it can increase complications such as ventilator‐related lung injury (VILI), ventilator atrophy, ventilator‐mediated diaphragmatic dysfunction (VIDD) (Laghi & Shaikh, 2014), and reduce the quality of life of patients and prolong hospital stay. At present, many parameters and tests are used to determine preparation for offline and extubation, including breathing rate, minute ventilation (MV), shallow fast breathing index, primary breathing test(SBT), T‐tube test, and balloon leak test. A series of retrospective studies have shown that these indicators cannot alone predict successful withdrawal. However, there are certain limitations and re‐intubation rates, and the above methods are challenging to apply in infants. At the same time, most traditional ventilation modes are triggered by pressure or flow. However, these triggering methods will be caused by air leakage (Moerer et al., 2008), improper trigger sensitivity setting (Sinderby et al., 1999), the need to overcome endogenous positive end‐expiratory pressure (PEEP) (Esquinas, 2013), and other problems, leading to delayed triggering and/or false triggering, increasing the work of the respiratory muscles of the patient, causing fatigue of the respiratory muscles, and adversely affecting the patient's prognosis (Purro et al., 2000).

Therefore, it is critical to assess whether children with ICU mechanical ventilation have ventilator weaning conditions. We urgently need a new ventilation mode to change this situation. Studies found that (Beck et al., 2000 , 2006; Finucane et al., 2005) electromechanical activities can be used to study diaphragm function, laying a foundation for the generation of mechanical ventilation triggered by EAdi signals. Neurally adjusted ventilatory assist (NAVA) is a new ventilation mode in which the ventilator is controlled by the electrical activity of the diaphragm (EAdi). It is suitable for any mechanical ventilation patients with spontaneous breathing by monitoring the EMG activity of the diaphragm (Slutsky & Brochard, 2005). NAVA ventilation is to collect EAdi signal and sense diaphragm action potential by placing diaphragmatic electrode catheter through nose and lower esophagus; transmit EAdi signal to ventilator equipped with Nava software through wire to complete diaphragm action potential transmission; ventilator computer receives, converts and analyzes signals, sends out ventilation instructions, and implements nerve ventilation coupling (Stein & Firestone, 2014). Based on the above principles, NAVA is synchronous and efficient; the patient's spontaneous breathing determines the respiratory rate and cycle; through real‐time monitoring of EAdi frequency and intensity, NAVA ventilator provides real‐time respiratory support that changes according to the respiratory frequency and intensity signals emitted by the patient's central nervous system and keeps synchronization in real time (Ouattara, 2012).

Previous studies have shown that the NAVA model can be used in neonates and premature infants, and it is safe, effective and well tolerated (Stein & Firestone, 2014). However, compared with the current traditional mechanical ventilation model (CPAP), its safety, effectiveness, and prognosis of patients are low. At present, there are only small sample surveys at home and abroad. We need further research to provide a theoretical basis for later clinical treatment. In this experiment, we will compare the differences between Nava mode mechanical ventilation and traditional ventilation mode (CPAP) after regular spontaneous breathing in 25 children with PICU mechanical ventilation and explore the application of neurally adjusted ventilatory assist in infant ventilator weaning.

## 2 METHODS

### 2.1 Patients

This study included 25 infants with mechanical ventilation in PICU. The underlying diseases included severe pneumonia, acute respiratory distress syndrome, fulminant myocarditis, purulent meningitis, myasthenia gravis, and Guillain Barre syndrome (Group TPALICC, 2015). Exclusion criteria for this study were as follows: (1) children without spontaneous breathing after mechanical ventilation, (2) paraplegia, (3) severe diaphragmatic paralysis, (4) upper gastrointestinal hemorrhage, and (5) esophageal‐gastric varices bleeding. All procedures performed in our study involving human participants were in accordance with the ethical standards. The study was approved by the Ethics Committee of Maternal and Child Health Care Hospital of Hubei Province. Moreover, informed consent was obtained from all individual participants included in the study. Two infants could not be converted to NAVA mode without EAdi signal and were not included in the trial. There were 11 boys and 12 girls, ranging in age from 0.1 to 5.5 years old, with a mean age of 1.88 ± 1.48 years old, and the average body weight was 10.43 ± 4.55 kg. The basic situation of children is shown in Table 1.

**TABLE 1:** The basic situation of children

Patient characteristics		*n* (%)
Gender	Male	11 (47.83%)
	Female	12 (52.17%)
Age	≤1	10 (43.48%)
	>1	13 (56.52%)
Disease type	Severe pneumonia	9 (39.13%)
	ARDS	5 (21.74%)
	Purulent meningitis	4 (17.39%)
	Guillain‐Barre syndrome	2 (8.69%)
	fulminant myocarditis	1 (4.35%)
	ICU acquired myasthenia	1 (4.35%)
	myasthenia gravis	1 (4.35%)

Abbreviation: ARDS, acute respiratory distress syndrome; ICU, intensive care unit.

### 2.2 Methods

Twenty‐three patients received mechanical ventilation with NAVA mode after improving basic diseases, regular spontaneous breath and before the ventilator was withdrawn. After the operation, all children were mechanically ventilated with a servo I (MAQUET critical care, Solna, Sweden) ventilator, and the ventilation mode was pressure regulated volume control (PRVC). During the anesthesia and awake breathing exercise, a diaphragmatic electrical signal catheter (EAdi catheter) was inserted into nasal cavity to obtain the EAdi signal of diaphragm myoelectric activity. Then, CPAP and NAVA ventilation were given to each child 1 h before ventilator weaning, respectively. The support level of CPAP and NAVA, which is the setting of pressure level and NAVA level, reached the standard of tidal volume (VT) of 6–8 ml/kg, pressure support of 6–10 cm H_2_O, and NAVA level of 0.5–2.0 cm H_2_O/μV. The positive end‐expiratory pressure (PEEP) level and FiO_2_ level of the same patient remained unchanged of 6 cm H_2_O and 30%.

Record and monitor the following indicators after 30 min of ventilation at the beginning of each mode: (1) hemodynamics: monitoring heart rate (HR), mean arterial pressure (MAP), central venous pressure (CVP), and impedance cardiography monitoring cardiac index (CI). (2) Respiratory mechanics: respiratory function monitor monitoring peak inspiratory pressure (Ppeak), mean airway pressure (Pmean), and dynamic lung compliance (cdyn). (3) Pulmonary gas exchange: arterial blood was extracted, and pH, arterial oxygen (PaO_2_), partial pressure of carbon dioxide (PaCO_2_), and oxygen saturation (SaO_2_) were measured. (4) Respiratory muscle load: EAdi peak value and EAdi low value.

### 2.3 Statistics analysis

The data were analyzed with SPSS ll.5 (Chicago, IL, USA) for medical statistics. Count data were expressed as mean ±SD. Parameter statistics used paired *t* test and independent *t* test. Statistical significance was set at *p *< .05 level.

## 3 RESULT

Two of the 25 children were unable to switch to NAVA mode due to weakness in the respiratory muscles and no EAdi signal. There was one child with glycogenosis type II, one child with myasthenia gravis that caused weaning failure, and tracheotomy was performed.

Re‐tracheal intubation after ventilator withdrawal and re‐weaning after mechanical ventilation were successful in five children, including Guillain‐Barre syndrome, purulent meningitis, ICU‐acquired myasthenia, acute respiratory distress syndrome (ARDS), and myasthenia gravis. Analysis of the peak EAdi signal before NAVA ventilation and the peak EAdi signal after the second intubation in five children with failed intubation and secondary intubation were 11.14–3.6 cm H_2_O/μV and 30.04–8.4 cm H_2_O/μV, the difference is statistically significant (*p* < .01). The results are shown in Table 2.

**TABLE 2:** The comparison of various indicators of CPAP and neurally adjusted ventilatory assist (NAVA) modes in 23 patients

Factor	CPAP	NAVA	*t*	*p*‐Value	Correlation coefficient
HR	116.348 ± 20.054	115.739 ± 21.286	0.177	.861	0.685
MAP (mmHg)	70.130 ± 7.497	73.391 ± 7.488	−3.149	.005*	0.780
CVP (mmHg)	7.696 ± 1.608	7.304 ± 1.428	1.251	.224	0.517
CI (ml/min/m^2^)	4.500 ± 0.743	4.535 ± 0.722	−0.331	.744	0.763
Ppeak (cmH_2_O)	16.783 ± 1.976	13.783 ± 1.204	8.307	<.01*	0.495
Pmean (cmH_2_O)	10.913 ± 1.952	8.435 ± 1.037	6.492	<.01*	0.379
Cydyn compliance	6.065 ± 2.007	6.517 ± 1.965	−2.382	.026*	0.895
PO_2_ (mmHg)	121.435 ± 26.858	121.696 ± 24.771	−0.122	.904	0.924
PCO_2_ (mmHg)	37.304 ± 6.911	36.957 ± 5.287	0.252	.804	0.436
Asynchronous index	22.3 ± 3.8	0	12.892	<.01*	0.402

Abbreviations: CI, cardiac index; HR, heart rate; CVP, central venous pressure; MAP, mean arterial pressure.

* Indicates a significant correlation between before and after treatment (CPAP vs. NAVA).

### 3.1 Comparison of indicators of CPAP and NAVA models

The comparison of various indicators of CPAP and NAVA modes in 23 patients is shown in Table 3. Hemodynamic indexes, including CVP, were not statistically different between the two groups of CPAP and NAVA. PCO_2_ is not significantly different in the two modes, both were at normal levels, the peak inspiratory pressure (PIP) in NAVA mode is lower than that in CPAP mode (*p* < .05), and its EAdi signal was correspondingly low, which were (8.24–2.6) 1.1 V and (7.04–1.6) 1.1 V (*p* < .05). In addition, as far as the asynchronous index (AI) is concerned, the NAVA mode is also significantly lower than the CPAP mode (*p* < .01). There were statistically significant differences in ventilator peak inspiratory pressure (Ppeak), mean inspiratory pressure (Pmean), and compliance and mean arterial pressure (*p* < .01). The application time of NAVA mode is 24–240 h, with an average of 43.84–28.7 h.

**TABLE 3:** Comparison of Edi peak value and Edi low value

Edi value	Success	Failure	*t*	*p*‐Value
Edi peak value	18	5		
	4.654 ± 1.033	4.380 ± 1.117	0.001	.974
Edi low value	18	5		
	1.323 ± 0.711	1.920 ± 0.756	0.091	.766

## 4 DISCUSSION

For patients with a critical illness, successful ventilator weaning may be a stressful process experienced by the body. Its success depends on the relationship between multiple organ function stability and dysfunction. It appears that ventilator weaning failures are often the result of the multi‐layered dysfunction of lung, muscle, nerve, heart, and brain. Other factors such as metabolic disorders, malnutrition, and uncontrolled infections are equally important (Tang et al., 2013).

Mechanical ventilation is a supportive method for children with PICU respiratory failure. Basic lung injury and man–machine counteraction, improper use of ventilator, and ventilator‐related lung injury further aggravate respiratory failure, prolong hospital stay, and increase mortality. It is extremely important to synchrony of patient‐ventilator, reduce ventilator‐related lung injury, and select ventilator mode when working with patients. Premature or delayed weaning, mechanical ventilation‐induced lung injury will seriously affect patients’ prognosis (Chen et al., 2016). Patients with CPAP ventilation would significantly increase their breathing frequency and the work of respiratory muscles when their thoracic compliance changed (Bersten et al., 1989; Branson et al., 1994). Moreover, it was found that the incidence rate of the asynchronous index (AI) in the PSV mode was 36%, which was significantly higher than that in the NAVA mode. These problems will reduce the comfort of mechanical ventilation and increase dependence on sedatives (Bonacina et al., 2019). The results showed that the asynchronous index of Nava model was significantly lower than that of CPAP mode.

Compared with the traditional mechanical ventilation mode, NAVA can more effectively realize synchronization of time and support between the ventilator and the nerve center. NAVA relies on EAdi to trigger ventilator ventilation and complete the mutual conversion between inhalation and exhalation, and provides pressure support proportional to the intensity of EAdi. In theory, it can effectively synchronize the physiological ventilation time with the ventilator ventilation time (time synchronization). At the same time, the intensity of physiological inhalation and the support strength of the ventilator are matched (the support strength is synchronized). These have been confirmed in animal experiments and clinical studies.

NAVA is an assisted ventilation mode in which both inspiratory triggering and exhalation switching are determined by the changes in EAdi, which changes the history of the original assisted breathing mode triggered by changes in the airway mechanics. NAVA can give ventilation assistance in proportion according to the strength of EAdi signal, which theoretically minimizes the occurrence of trigger delay and human–machine dissonance. Studies (Piquilloud et al., 2011) in healthy volunteers have shown that NAVA can safely and effectively unload the respiratory muscles during maximal inspiratory effort without causing excessive lung dilation and expiration switching failures. Our results also show that patients with NAVA require significantly lower PIP than CPAP, indicating that NAVA has a greater potential to reduce lung injury than CPAP. Twenty‐three children with successful weaning used CPAP and NAVA modes. There were statistically significant differences in ventilator peak pressure (Ppeak), mean pressure (Pmean), and compliance, suggesting that NAVA ventilation mode is more suitable for patients' physiology and lower airway pressure. It can reduce ventilator‐related barotrauma, and there is a statistical difference in the mean arterial pressure, which corresponds to the mean pressure lower than the NAVA ventilation phase, which indicates that the NAVA ventilation chest pressure is lower and the hemodynamic effect is smaller. NAVA is Conducive to the recovery of heart and lung function in children (Sinderby et al., 2007). Beck et al. (2009) sequentially applied conventional mechanical ventilation and NAVA to seven low birth weight infants before and after extubation and compared the coordination between EAdi and pressure support level in these two ventilation modes. The results showed that NAVA relied on EAdi signal to perceive the drive of respiratory center of infants, which improved the synchrony of human and machine more than traditional ventilation mode, and was consistent with the results of this study. Breatnach et al. (2010) reported that in 16 children aged between two days and four years who needed mechanical ventilation, the peak airway pressure decreased by 28% after 30 min of NAVA ventilation and 32% after 3 h.

In patients with acute respiratory failure, there was a significant negative correlation between EAdi and stress support which means as pressure support increases, EAdi decreases proportionally. It is indicated that the strength of EAdi is an indicator of the respiratory load. Since the acquisition of EAdi signals plays a crucial role in NAVA, it can be used for neuromuscular diseases to evaluate whether the muscle function is restored and choose a more appropriate offline time. In this trial, the peak Edi of children who failed to withdraw was significantly different from that after withdraw. Whether this result can indicate that higher EAdi reflects better diaphragmatic function and respiratory drive, which lead to a higher success rate of weaning, but EAdi predicts whether the threshold of successful weaning exists, and whether infants and young children of different ages have differences to be further explored. It can be suggested that a higher EAdi reflects a better diaphragmatic function and respiratory drive, which leads to a higher rate of successful weaning, but EAdi predicts whether there is a threshold for successful withdrawal, and whether there are differences between infants of different ages needs further exploration. Besides, the experimental samples are difficult to obtain, so there are some limitations in the study, and we still need to expand the samples for further in‐depth study

## 5 CONCLUSION

NAVA that has the same safety in hemodynamics can reduce the occurrence of lung injury in comparison with the traditional CPAP model and predict whether children have the condition of ventilator off‐line. NAVA ventilation, a new ventilation mode, can maximize human–machine coordination while ensuring that the ventilator supports the appropriate ventilation level for patients, reducing complications related to mechanical ventilation, protecting organ functions, improving respiratory mechanics and hemodynamics. Guiding the successful evacuation of ventilator can improve the survival rate, reduce the time of mechanical ventilation, and reduce the medical cost.

## CONFLICT OF INTEREST

The authors declare no conflict of interest.

## AUTHOR CONTRIBUTIONS

Shuna Xiao and Hui Xu contributed to the conception and design of the study. Chengjiao Huang, Ying Cheng, Zhi Xia, Yong Li, Wen Tang, Buyun Shi, and Lijun Wang performed the experiments. Xiaolan Shu, Ying Jiang, and Chenguang Qin collected and analyzed the data. Shuna Xiao and Hui Xu wrote the manuscript. All authors reviewed and approved the final version of the manuscript.

### PEER REVIEW

The peer review history for this article is available at https://publons.com/publon/10.1002/brb3.2350

## Data Availability

The datasets generated and analyzed during the current study are available from the corresponding author on reasonable request.
